# Purified Tea (*Camellia sinensis* (L.) *Kuntze*) Flower Saponins Induce the p53-Dependent Intrinsic Apoptosis of Cisplatin-Resistant Ovarian Cancer Cells

**DOI:** 10.3390/ijms21124324

**Published:** 2020-06-17

**Authors:** Ning Ren, Lianfu Chen, Bo Li, Gary O. Rankin, Yi Charlie Chen, Youying Tu

**Affiliations:** 1 Department of Tea Science, Zhejiang University, 866 Yuhangtang Road, Hangzhou 310058, China; ningren@zju.edu.cn (N.R.); c.lianfu@foxmail.com (L.C.); drlib@zju.edu.cn (B.L.); 2 College of Health, Science, Technology and Mathematics, Alderson Broaddus University, 101 College Hill Drive, Philippi, WV 26416, USA; 3 Department of Biomedical Sciences, Joan C. Edwards School of Medicine, Marshall University, Huntington, WV 25755, USA; rankin@marshall.edu

**Keywords:** tea flower saponins, Chakasaponin I, ovarian cancer, intrinsic apoptosis, p53 pathway

## Abstract

Ovarian cancer is currently ranked at fifth in cancer deaths among women. Patients who have undergone cisplatin-based chemotherapy can experience adverse effects or become resistant to treatment, which is a major impediment for ovarian cancer treatment. Natural products from plants have drawn great attention in the fight against cancer recently. In this trial, purified tea (*Camellia sinensis* (L.) *Kuntze*) flower saponins (PTFSs), whose main components are Chakasaponin I and Chakasaponin IV, inhibited the growth and proliferation of ovarian cancer cell lines A2780/CP70 and OVCAR-3. Flow cytometry, caspase activity and Western blotting analysis suggested that such inhibitory effects of PTFSs on ovarian cancer cells were attributed to the induction of cell apoptosis through the intrinsic pathway rather than extrinsic pathway. The p53 protein was then confirmed to play an important role in PTFS-induced intrinsic apoptosis, and the levels of its downstream proteins such as caspase families, Bcl-2 families, Apaf-1 and PARP were regulated by PTFS treatment. In addition, the upregulation of p53 expression by PTFSs were at least partly induced by DNA damage through the ATM/Chk2 pathway. The results help us to understand the mechanisms underlying the effects of PTFSs on preventing and treating platinum-resistant ovarian cancer.

## 1. Introduction

Ovarian cancer is currently ranked at fifth in cancer deaths that affect females [1]. According to the American Cancer Society estimates, about 13,940 ovarian cancer patients will die among 21,750 new women patients in the United States in 2020 [1]. The chemotherapy resistance and the inability to detect ovarian cancer in its earliest stages are the biggest problems that doctors face today [2]. Cisplatin is widely used as a chemotherapy medicine to treat ovarian cancer. However, it might cause tumor recurrence and chemotherapy resistance in patients, which are the main reasons for treatment failure in most patients with metastatic disease [3]. In ovarian cancer, key apoptotic regulators, such as the Akt family, p53, and death-receptor family, mediate the cell response to cisplatin, which plays a vital role in the induction of resistance of cancer cells to chemotherapeutic agents [4]. It was reported that the over-expression of anti-apoptotic Bcl-2 family members, including Bcl-2 and Bcl-xL or down-regulation or mutation of pro-apoptotic factors, such as Bax and caspases, are associated with platinum-based chemotherapy resistance [5]. Facing this problem, researchers have been looking over new and more unique ways for cancer therapy. It is important to find new cancer treatments that cause fewer adverse effects for patients [6,7]. Natural products from plants have gotten great interests in the fight against cancer. Tea, as a worldwide popular beverage made from the *Camellia sinensis* (L.) *Kuntze* plant leaves, has been studied thoroughly for its beneficial biofunctions, including cancer prevention. The relationship between human cancer risk and tea consumption have been reviewed [8]. Studies suggested that tea consumption exerts an inhibitory influence at all stages of lung carcinogenesis [9,10,11] and also on chemically induced oral [12], oesophageal [13] and gastric [14] carcinogenesis. Tea polyphenols were studied sufficiently for their functions in cancer prevention [15], and tea seeds’ saponins also attracted researchers’ attention in recent years and were reported to have anti-cancer effects by several studies [16,17,18].

Tea (*Camellia sinensis* (L.) *Kuntze*) flowers have quite similar chemical components compared with tea leaves [19]. The anti-cancer effects of tea leaves, as mentioned above, have been widely studied, but fewer studies have been performed on the health benefits of the tea flowers. Being an abundant agricultural by-product in China and Japan, tea flowers have attracted greater attention as natural products containing rich bioactive compounds in recent years. Several reports showed that tea flower extracts presented potential anti-cancer activities in vitro [20,21]. Our previous study found that an extract from flowers of the tea variety Longjing 43 has very low acute and sub-chronic toxicity and no mutagenic potential towards rats [22]. Triterpenoid saponins are characteristic ingredients and have a higher content in tea flowers compared with that in tea leaves. We previously identified 21 triterpenoid saponins in extracts from tea flowers in selected cultivars [23]. In recent years, the bio-functional effects of tea flower saponins such as antihyperlipidemic, antihyperglycemic, anti-obesity, anti-cancer, and gastroprotective effects have been reported [24,25,26], among which Chakasaponin I has been reported to have antihyperlipidemic and antihyperglycemic [27], anti-cancer [26], and anti-obesity [28] effects. We previously reported that tea flower saponins containing 14 triterpenoid saponins could induce cell cycle arrest and apoptosis in human ovarian cancer cells [29].

Apoptosis is a genetically encoded cell death process that can be triggered by intrinsic and extrinsic pathways [30,31,32]. The failure to induce apoptosis is one of the underlying causes of drug resistance, and its molecular mechanisms in ovarian cancer cells include abnormal expression of the Bcl-2 family proteins, p53 mutations, upregulation of other inhibitors of apoptosis which block activation of caspases and stabilize the mitochondrial transmembrane potential. Thus, triggering apoptosis is one of the promising strategies to overcome the chemo-resistance of cancer cells [33,34]. A recent case reported that the splice mutation in *TP53* was present as an early driver mutation at diagnosis in high-grade serous ovarian cancer [35]. The association between gain-of-function mutations in *TP53* and patient sensitivity to platinum-based treatment was also observed in high-grade serous ovarian cancer [36]. DNA damage in a cell could activate the p53 protein. There are many chemical and physical causes of DNA damage, including alkylation of bases, UV irradiation, cross-linking and depurination of DNA, and more [37]. Each type of DNA damage is diverse and repaired by different enzymes in diverse ways. However, most damages to the integrity of DNA in a cell could activate the p53 protein, which means every kind of DNA damage is related with the p53 protein and its pathway [37,38,39,40].

In the present study, we isolated and purified tea flower saponins (PTFSs), which mainly contain Chakasaponin I and Chakasaponin IV. The anti-cancer effects and the underlying mechanisms of PTFSs on human endothelial ovarian cancer cell lines A2780/CP70 and OVCAR-3 and human normal ovarian epithelial cell line IOSE-364 were investigated.

## 2. Results

### 2.1. Chemical Composition of PTFSs

**Figure 1:**
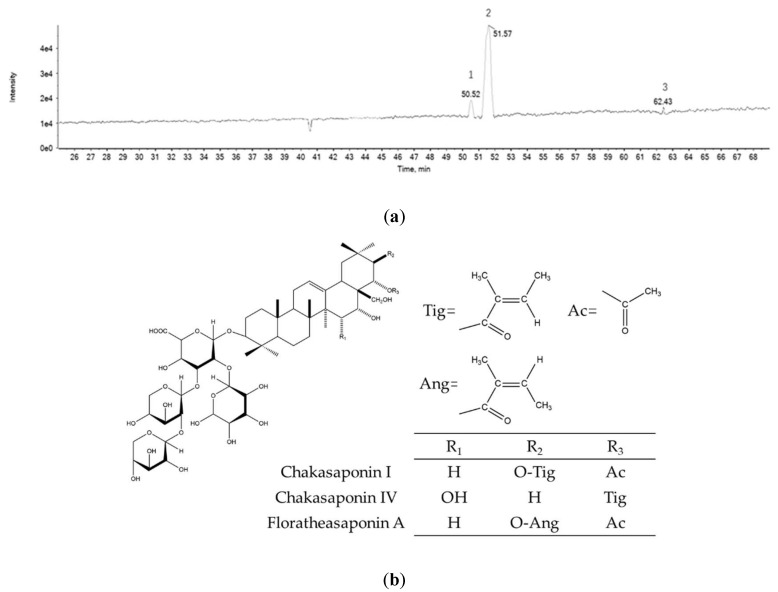
Characteristic of purified tea flower saponins (PTFSs). (**a**) Base peak chromatogram of PTFSs. (**b**) Chemical structures of Chakasaponin I, Chakasaponin IV and Floratheasaponin A [25,26].

Purified tea flower saponins (PTFSs) were successfully characterized by ultra-performance liquid chromatography coupled with electrospray ionization quadrupole time-of-flight mass spectrometry (UPLC-Q-TOF/MS/MS) analysis. Three main bioactive compounds were found to be present (Figure 1a). The molecular formulas of these saponins were identified by high-resolution mass measurements, deprotonated molecular ions [M-H]^−^, and fragment ions (Table 1). The three saponins were Chakasaponin IV, Chakasaponin I and Floratheasaponin A (Figure 1b) (allocating 8.1%, 80.1%, and 1.4%, respectively), based on published fragmentation data and nominal mass calculated from known structures [23,25,27].

### 2.2. PTFSs Inhibit Cell Viability and Cell Proliferation in A2780/CP70s and OVCAR-3s

**Figure 2:**
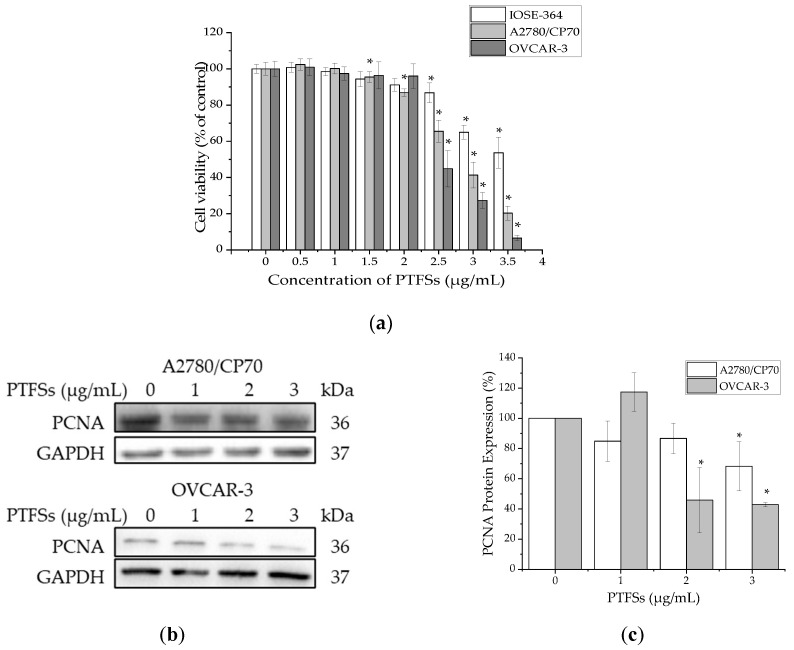
Cytotoxicity and proliferation inhibition effects of PTFSs on A2780/CP70, OVCAR-3 and IOSE-364 cells. (**a**) Cell viability, 24 h after treatment with different concentrations of PTFSs (0–3.5 μg/mL), was examined using the MTS assay. (**b**,**c**) Effects of PTFSs (0, 1, 2, 3 μg/mL) on the expression of proliferating cell nuclear antigen (PCNA) were determined by Western blotting. GAPDH was utilized for an endogenous reference to standardize protein levels. All values were expressed as mean ± standard error of mean (SEM) of three independent experiments. * *p* < 0.05 versus control.

The cytotoxicity of PTFSs on A2780/CP70s, OVCAR-3s and IOSE -364s was analyzed using the MTS assay. Cells were treated with various concentrations (0–3.5 μg/mL) of PTFSs for 24 h. As shown in Figure 2a, PTFSs inhibited the growth of all three cell lines in a dose-dependent manner, but had a lower cytotoxic effect against normal epithelial ovarian cell line IOSE-364 cells than that against ovarian cancer cell lines A2780/CP70 and OVCAR-3. The cell viability decreased from 100% to 20.28% for A2780/CP70, from 100% to 6.52% for OVCAR-3, and from 100% to 53.64% for IOSE-364 cells after being treated with PTFSs (0–3.5 μg/mL) for 24 h. The IC_50_ of PTFSs was 2.71 µg/mL for A2780/CP70, 2.58 µg/mL for OVCAR-3, and 3.90 µg/mL for IOSE-364. To verify whether PTFSs inhibit cell proliferation, we also checked the protein expression of proliferating cell nuclear antigen (PCNA) in both ovarian cancer cell lines by Western blotting. We found that PTFSs significantly decreased its expression in the treatment group compared with control group (Figure 2b,c). These results confirm that PTFSs inhibit cell viability and cell proliferation in ovarian cancer cells.

### 2.3. PTFSs Induce Apoptosis in A2780/CP70s and OVCAR-3s 

**Figure 3:**
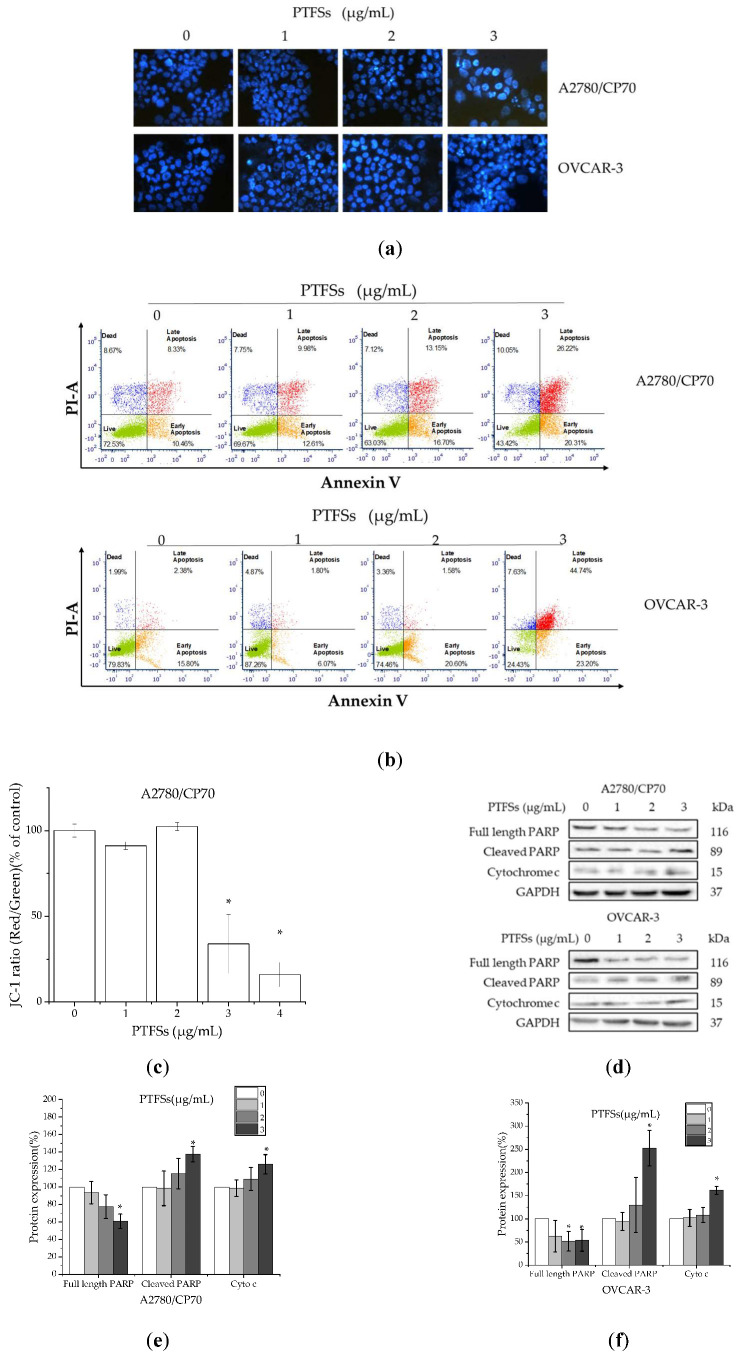
PTFSs induced apoptosis in A2780/CP70 and OVCAR-3 cells. (**a**) Cells stained with Hoechst 33342 were detected by fluorescent microscopy (magnification, ×400), after being treated with various concentrations of PTFSs for 24 h. The bright nuclei represent apoptotic cells. Intact nuclei represent viable cells. (**b**) Cells treated with various concentration PTFSs were detected by flow cytometry using a double-staining method with FITC-conjugated Annexin V and PI. (**c**) Quantification histograms represent the ratio of JC-1 aggregates to JC-1 monomers (ratio of 590:530 nm emission intensity) of A2780/CP70 cells, which reveals ΔΨm dissipation after 24 h treatment with various concentrations PTFSs. (**d**–**f**) Protein levels of full length PARP, cleaved PARP, and cytochrome c were detected by Western blotting. GAPDH was utilized for an endogenous reference to standardize protein levels. All data were expressed as mean ± SEM of three independent experiments. * *p* < 0.05 compared with the control group. Above four treatments concentration of PTFSs were 0, 1, 2, and 3 μg/mL respectively.

To study if PTFSs inhibit cell viability and proliferation through inducing apoptosis in ovarian cancer cells, the changes in nuclear morphology of A2780/CP70s and OVCAR-3s treated with PTFSs (0,1,2,3 μg/mL) for 24 h were analyzed by the Hoechst 33342 DNA staining and observed under a fluorescence microscope. As shown in Figure 3a, 24 h PTFS treatment could cause obvious chromatin condensation and nucleus shrinkage in A2780/CP70s and OVCAR-3s. Such results are further verified by the results of flow cytometry assay. The results show that PTFSs significantly decreased the live cell percentage and increased the apoptotic cell one in both ovarian cancer cell lines in a concentration-dependent manner (Figure 3b). The total apoptotic cells increased from 19.53% to 48.34% among A2780/CP70 cells and increased from 18.53% to 66.03% among OVCAR-3 cells. In addition, the JC-1 aggregates/JC-1 monomers ratio in A2780/CP70 cells was quantitated by fluorescence spectrophotometer. The results show that the mitochondrial membrane potential collapsed after being treated with 3 and 4 μg/mL PTFSs for 24 h (Figure 3c), which suggested the early appearance of apoptosis. We further checked the protein levels of cleaved PARP and cytochrome c, which served as a marker and executor during cell apoptosis, respectively [31,37,41]. The results show that PTFSs increased the protein levels of cleaved PARP and cytochrome c in A2780/CP70s and OVCAR-3s, and the protein level of full length PARP were decreased (Figure 3d–f). All above data suggest that PTFS treatment induced mitochondrial membrane potential collapsing and apoptosis in ovarian cancer cells, and the inhibition of PTFSs on ovarian cancer cell viability might be due to inducing apoptosis.

### 2.4. PTFSs Induce Apoptosis via Caspase-3/7 and -9 Activation in A2780/CP70s

**Figure 4:**
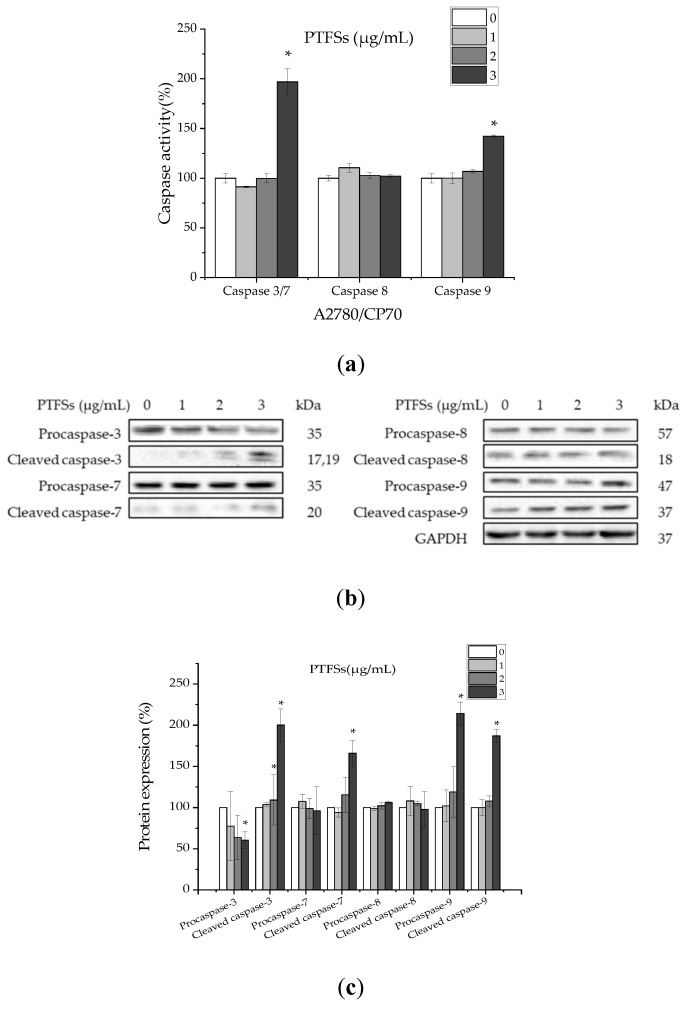
The effect of PTFSs on caspase activities in A2780/CP70 cells. (**a**) Cells were treated with PTFSs (0, 1, 2, and 3 μg/mL) for 24 h and the activities of caspase-3/7, -8 and -9 were determined. The caspase 3/7, -8, -9 activities of control cells were expressed as 100%. (**b**,**c**) Effects of PTFSs (0, 1, 2, 3 μg/mL) on the expression of caspase-3, -7, -8 and -9 were assayed by Western blotting. GAPDH was utilized for an endogenous reference to standardize protein levels. All values are shown as mean ± SEM of three independent experiments. * *p* < 0.05 versus control. Cells treated with culture medium containing 0.01% dimethyl sulfoxide (DMSO) was used as the control.

Caspases, as a family of cysteine proteases, play an important role in apoptotic responses. To demonstrate whether the PTFS-induced apoptosis in ovarian cancer cells was associated to the activation of caspases, we examined the activities of caspase-3/7, -8 and -9 in A2780/CP70 cells after being treated with PTFSs for 24 h, as shown in Figure 4a. Treatment with PTFSs enhanced the caspase-3/7 and -9 activities by 1.97- and 1.42-fold compared to that in controls, respectively, while it did not affect the activity of caspase-8. In addition, the protein expression of caspase-3, -7, -8 and -9 were also examined by Western blotting (Figure 4b,c). The results show that the protein expressions of cleaved caspase-3, -7 and -9, and procaspase-9 were significantly increased; contrarily, that of procaspase-3 was decreased, while the protein expressions of procaspase-7, -8 and cleaved caspase-8 were not affected after treated with PTFSs. All the above data suggest that PTFS-induced apoptosis is related to the activation of caspase-3, -7 and -9, but not related to the activation of caspase-8. 

### 2.5. PTFSs Induce Apoptosis through the Intrinsic Rather than Extrinsic Apoptotic Pathway in A2780/CP70s 

**Figure 5:**
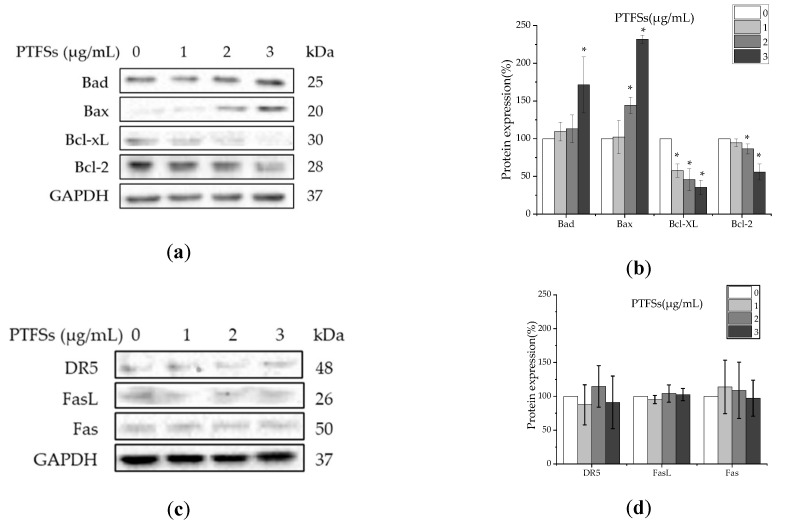
The effects of PTFSs on key proteins in intrinsic and extrinsic apoptotic pathways in A2780/CP70 cells. (**a**) Cells were treated with PTFSs (0, 1, 2, 3 μg/mL) for 24 h. Intrinsic apoptotic pathway-related proteins Bad, Bax, Bcl-xL, Bcl-2, and GAPDH protein expression were detected by Western blotting. (**c**) Cells were treated with PTFSs (0, 1, 2, 3 μg/mL) for 24 h. Extrinsic apoptotic pathway related proteins Fas, DR5, and FasL. (**b**,**d**) The changes in the protein levels induced by PTFSs were expressed as quantification histograms with error bars. GAPDH protein expression was detected by Western blotting and utilized for an endogenous reference to standardize protein levels. * *p* < 0.05 versus control. Cells treated with culture medium containing 0.01% DMSO were used as the control.

As caspase-8 and -9 are respectively the initiators of the extrinsic and intrinsic apoptotic pathway, we further investigated whether the intrinsic or extrinsic pathway participated in the PTFS-induced apoptosis of A2780/CP70s. We first checked the expressions of pro-apoptotic proteins Bax and Bad, and anti-apoptotic proteins Bcl-2 and Bcl-xL, which belong to the Bcl-2 families, related to the intrinsic apoptotic pathway. (Figure 5a,b). The results show PTFSs significantly upregulated Bax and Bad proteins, and downregulated Bcl-xL and Bcl-2 proteins. In other hand, we also examined the protein expression of extrinsic apoptotic pathway-related death receptors DR5, Fas, and FasL and the results show that PTFSs had no effects on these proteins (Figure 5c,d). These results suggest that PTFSs may induce apoptosis in A2780/CP70s through the intrinsic pathway rather than extrinsic pathway, which is accordant with the above results of caspase activities. 

### 2.6. PTFS-Induced Intrinsic Apoptosis Is p53-Dependent in Ovarian Cancer Cells

**Figure 6:**
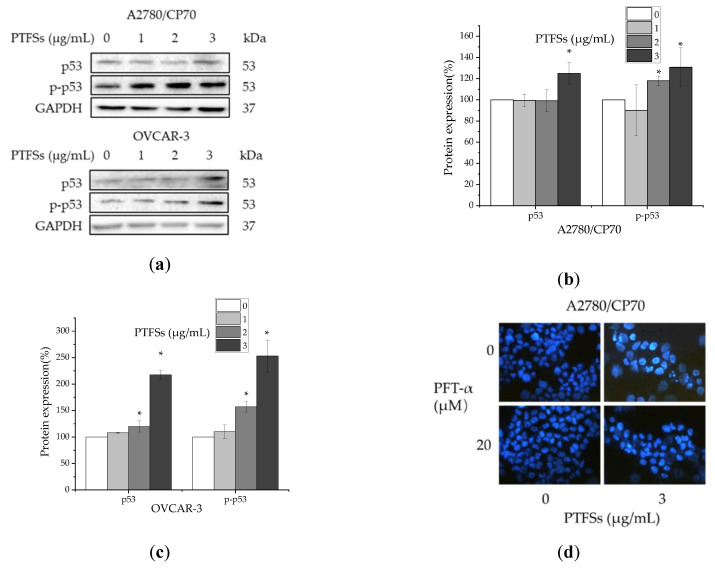

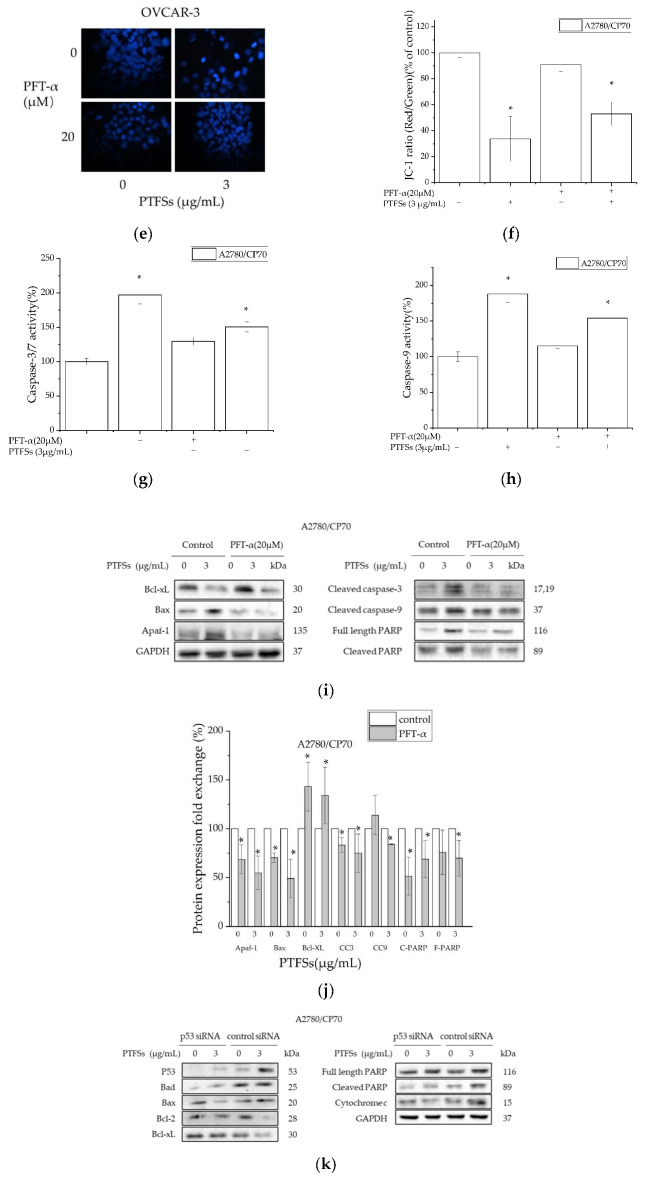

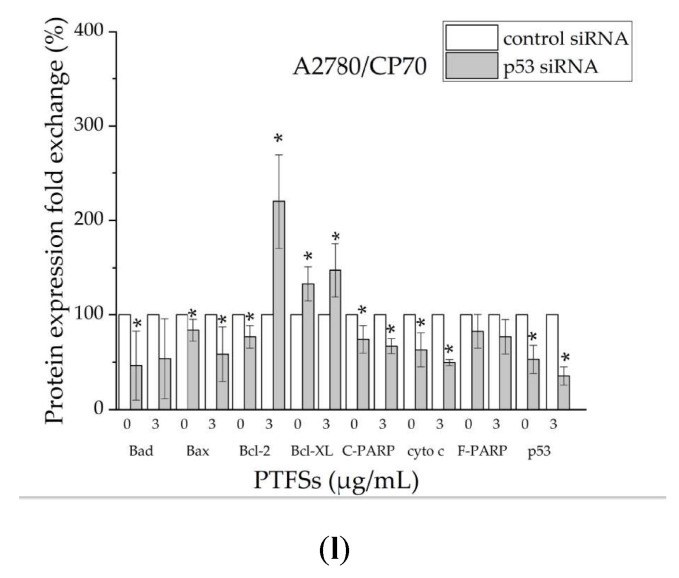
The changes in protein expressions in ovarian cancer cells apoptosis treated with PTFSs via p53 pathway. The quantification histograms are shown with error bars. Data represent means ± SD from three independent experiments. (**a**–**c**) The effects of PTFSs on the protein expression of phospho-p53 (Ser15) and p53 determined by Western blotting in A2780/CP70 and OVCAR-3 cells. (**d**,**e**) Cellular apoptosis was measured after cells were treated with PTFSs and p53 inhibitor PFT-α for 24 h and then stained with Hoechst 33342 in A2780/CP70 (**d**) and OVCAR-3 (**e**) cells (×400). (**f**) Bar charts represent the ratio of JC-1 aggregates to JC-1 monomers (ratio of 590:530 nm emission intensity) of A2780/CP70 cells, which reveals ΔΨm dissipation after 24 h treatment with or without PTFSs or p53 inhibitor PFT-α. (**g**,**h**) caspase 3/7 and caspase 9 activities were determined after treatment with PTFSs and p53 inhibitor PFT-α for 24 h in A2780/CP70 cells. (**i**,**j**) The effects of pre-incubation of PFT-α (20 μM) on the protein expression of Apaf-1, Bax, Bcl-XL, cleaved caspase-3 and -9, full length PARP and cleaved PARP determined by Western blotting in A2780/CP70 cells. (**k**,**l**) The effects of p53 siRNA (50 nM) on the protein expression of Bad, Bax, Bcl-2, Bcl-XL, full length PARP and cleaved PARP and cytochrome c determined by Western blotting in A2780/CP70 cells. GAPDH protein expression was detected by Western blotting and utilized for an endogenous reference to standardize protein levels. * *p* < 0.05, compared with respective controls. CC3, CC9, C-PARP, F-PARP, and cyto c are the abbreviations of cleaved caspase 3, cleaved caspase 9, cleaved PARP, full length PRAP, and cytochrome c, respectively.

PTFSs significantly increased the p53 protein expression and phosphorylation at Ser15 of p53 in our study (Figure 6a–c), which prompted us to explore the role that p53 played in PTFS-induced apoptosis in ovarian cancer cells. We firstly pre-incubated cells with p53 specific inhibitor pifithrin-α (PFT-α) (20 μM) for 24 h and performed the Hoechst 33342 and JC-1 assays after the PTFS treatment to check the cell apoptosis. The caspase activity assays were also performed to determine whether the activities of caspase-3/7 and -9 increased by PTFSs being alleviated by the pre-incubated with PFT-α. The results show that pre-incubation of PFT-α could decrease the apoptotic rate (Figure 6d,e) and JC-1 ratio (Figure 6f) and reverse the PTFS-induced activities of caspase-3/7 (Figure 6g) and caspase-9 (Figure 6h), which suggests that p53 is involved in PTFS-induced apoptosis in A2780/CP70 cells. Next, the results of western blotting also show the effects of pre-incubation of 20 μM PFT-α on the intrinsic apoptosis related protein levels in the cells treated with PARP (Figure 6i,j). The overexpression in A2780/CP70 cells of Apaf-1, Bax, cleaved caspase-3, cleaved caspase-9, full length PARP and cleaved PARP after PTFS treatment was decreased by PFT-α pre-incubation compared with the control group. Meanwhile, the inhibition of Bcl-xL expression in A2780/CP70 cells after the PTFS treatment was reversed by PFT-α pre-incubation compared with control group. 

Moreover, we knocked out p53 gene by p53 siRNA and then checked intrinsic apoptotic-related proteins. The results show that knockdown of p53 resulted in significant inhibition of p53 overexpression after PTFS treatment. This p53 depletion attenuated the decreasing by PTFS treatment of Bcl-2, and Bcl-xL protein expressions and reversed the PTFS-induced increasing of Bad, Bax and cytochrome c protein expressions. (Figure 6k,l) 

According to all the results above, it was suggested that p53 is a pivotal mediator of PTFS-induced intrinsic apoptosis in ovarian cancer cells. Meanwhile, it is also hinted that Bcl-2 family proteins, caspase family proteins, DNA damage-related protein p-Histone H2A.X, apoptosis executor cytochrome c and Apaf-1 were regulated in the process of PTFS-induced intrinsic apoptosis through p53 pathway in A2780/CP70 cells.

### 2.7. PTFSs Induce DNA Damage in Ovarian Cancer Cells

**Figure 7:**
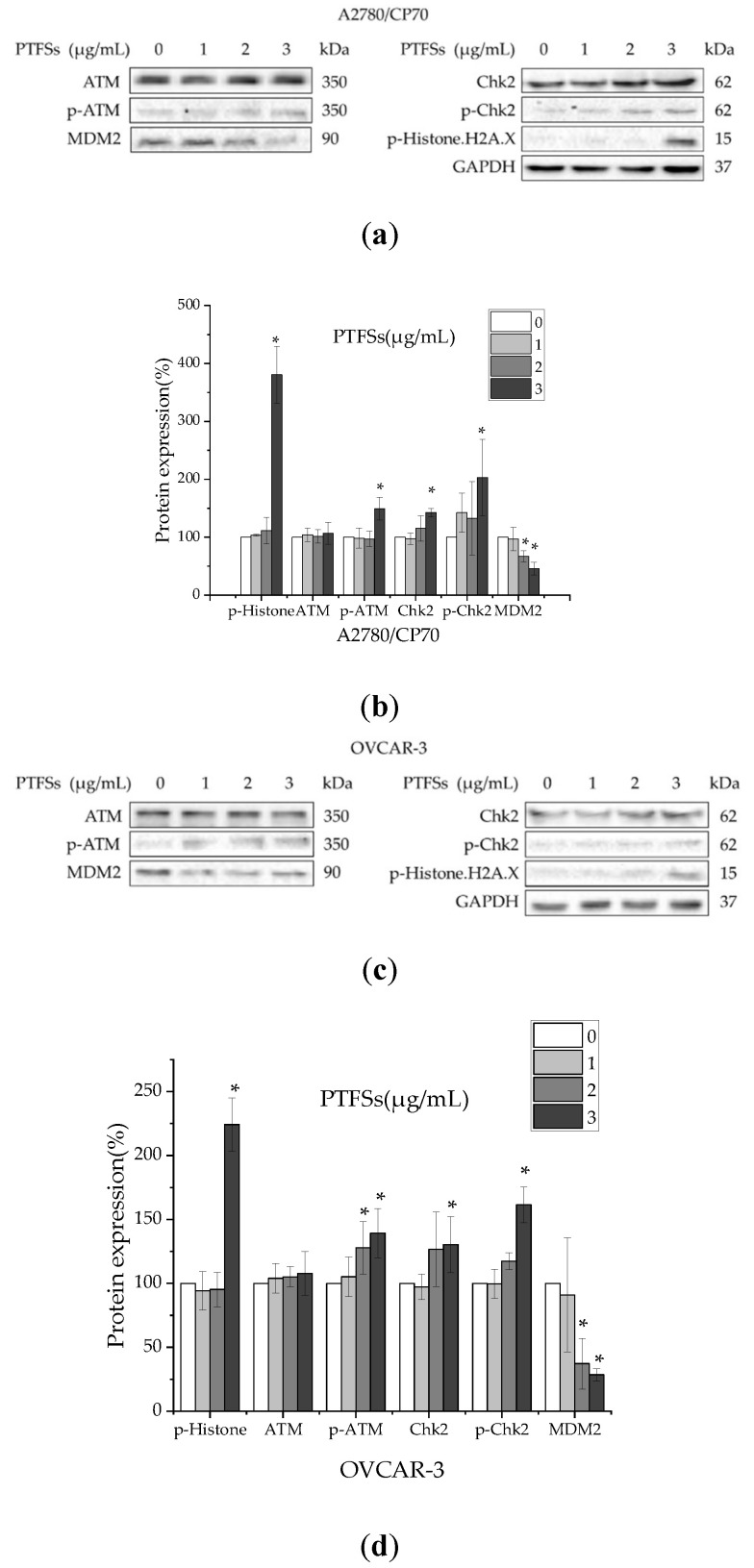
The changes in DNA damage-related proteins in ovarian cancer cells treated with PTFSs. (**a**,**c**) A2780/CP70 and OVCAR-3 cells were treated with PTFSs (0, 1, 2, 3 μg/mL) for 24 h. p-Histone H2A.X, ATM, p-ATM, Chk2, p-Chk2, MDM2 and GAPDH protein expression were detected by Western blotting. (**b**,**d**) The changes in the protein levels induced by PTFSs are expressed as quantification histograms with error bars in A2780/CP70 and OVCAR-3 cells. GAPDH was utilized for an endogenous reference to standardize protein levels. Results are expressed as mean ± SEM from three independent experiments. * *p* < 0.05 versus control. Cells treated with culture medium containing 0.01% DMSO was used as the control.

The p53 protein plays an important role response to DNA damage. To investigate the relationship of DNA damage and the up-regulation of p53 by PTFSs, DNA damage-related proteins, including p-histone H2A.X, ATM, p-ATM, Chk2, p-Chk2 and MDM2, were determined in A2780/CP70s and OVCAR-3s. The phosphorylation of histone H2A.X at Ser139 suggests DNA double-strand breaks. As shown in Figure 7, PTFSs significantly upregulated the protein expressions of p-histone H2A.X and p-ATM, but had no effect on the protein level of total ATM. Chk2 can be overexpressed in cancer cells and phosphorylated by p-ATM. PTFSs significantly increased the protein level of p-Chk2, reduced that of MDM2, but had no significant effect on that of total Chk2. These results suggest that PTFS treatment could induce DNA damage in both A2780/CP70s and OVCAR-3s. The p53 protein can be phosphorylated by ATM at Ser15, promoting both the activation and accumulation of p53 in response to DNA damage [42]. As PTFSs significantly increase the protein levels of p-p53 (Ser15) (Figure 5a), it could be reasonable deduced that PTFSs induce DNA damage by activating the ATM-Chk2 pathway, and sequentially up-regulate the p53 expression as well as phosphorylate p53 at Ser15.

## 3. Discussion

Ovarian cancer is a lethal gynecological cancer that affect females all around the world. Patients who have undergone cisplatin-based chemotherapy can experience adverse effects or become resistant to treatment, which is a major impediment for ovarian cancer treatment [43]. Natural products have received new interest as a rich source for drug discovery. Saponins have appeared as one of the most promising anti-cancer treatments, as shown by several recent studies [17,18,44,45]. The biofunctions of saponins isolated from the flower bud extracts of *C. sinensis* in Japan and China, and *C. sinensis var. assamica* in India have been reviewed [24]. Our previous work demonstrated that saponins isolated from tea (*Camellia sinensis* (L.) *Kuntze*) flowers which contain 14 triterpenoid saponins exclusive of Chakasaponin I and Chakasaponin IV can induce apoptosis in ovarian cancer A2780/CP70 cells [29]. In the present study, we firstly purified tea (*Camellia sinensis* (L.) *Kuntze*) flower saponins (PTFSs) which mainly consist of Chakasaponin I (80.1%) and Chakasaponin IV (8.1%) (Figure 1). PTFSs were tested for anti-cancer abilities in A2780/CP70 and OVCAR-3. We discovered that PTFSs powerfully inhibit cell viability and cell proliferation of A2780/CP70s and OVCAR-3s, and demonstrated that these inhibitory effects were likely due to the induction of intrinsic apoptosis by regulation of p53 protein expression.

The MTS assay showed that PTFSs were cytotoxic to A2780/CP70s and OVCAR-3s, with relatively lower cytotoxicity in the normal ovarian surface epithelial cell line IOSE-364 (Figure 2a). Expression and synthesis of PCNA were reported to be linked with cell proliferation [46,47,48]. PTFS treatment significantly decreased protein expression of PCNA of A2780/CP70s and OVCAR-3s (Figure 2b,c), which suggested the inhibition on the cell proliferation by PTFSs. The results are accordance with our previous study [29]. 

A potential mechanism of chemotherapy drug and radiation resistance in cells might be their resistance to apoptosis [49]. Brightly stained and condensed nuclei were considered to be apoptotic, which were shown by Hoechst 33342 staining in PTFS-treated A2780/CP70s and OVCAR-3s (Figure 3a). The results are in accordance with those from the flow cytometry assay, which show a significantly increased proportion of apoptotic cells in the PTFS-treated groups (Figure 3b). These results suggest that PTFSs induce apoptosis in ovarian cancer cells. In healthy cells, JC-1 is further concentrated within the mitochondria, stimulated through the mitochondrial transmembrane potential (ΔΨm), where it develops into red-emitting aggregates, compared to that in the cytosol, where it presents as a green-fluorescent monomer. Therefore, the red to green JC-1 fluorescence ratio may be utilized as a sensitive quantification of ΔΨm [50,51]. A reduction in red fluorescence and an increase in green fluorescence can signal the disruption of ΔΨm, which is generally thought to be a “point-of-no-return” in the actions that lead up to apoptosis [30]. Our results demonstrate that PTFS treatment decreased the JC-1 ratio (red/green), which leads to the dissipation of ΔΨm in A2780/CP70s (Figure 3c). Through permeabilizing the mitochondrial outer membrane, intermembrane space proteins, including the apoptotic signaling molecules cleaved PARP and caspase activator cytochrome c, are released into the cytosol [52]. We detected cleaved PARP and cytochrome c protein, which indicates that PTFS treatment significantly increases levels of both proteins and decreases expression of full length PARP (Figure 3d–f) in A2780/CP70s and OVCAR-3s. These data further confirm the stimulation of apoptosis in ovarian cancer cells by PTFS treatment.

Apoptosis could be separated into three separate phases including initiation, integration/decision and execution/degradation. Initiation is dependent on the type of the fatal signal, which can develop due to either the intracellular (intrinsic) microenvironment or the extracellular (extrinsic pathway) [50]. Caspases are initiators and executioners in apoptosis-related signaling pathways [53]. Initiator caspases include caspase-8, which is necessary for the extrinsic apoptosis pathway, caspase-9 (necessary for the intrinsic one), and executioner caspases (i.e., caspase-3 and caspase-7) [54]. The effects of PTFSs on caspase-3/7, -8 and -9 in A2780/CP70 cells were investigated in our study. PTFSs increased the caspase-3/7 and -9 activities and had no effect on the activity of caspase-8 (Figure 4a). At the same time, we detected levels of procaspase-3, -7, -8, -9 and cleaved caspase-3, -7, -8, -9 proteins. The results indicate that PTFSs heighten cleaved caspase-3, -7, and -9, while having no influence on that of cleaved caspase-8 (Figure 4b,c). Caspase activities and Western blotting assay both demonstrated that PTFSs may stimulate apoptosis in A2780/ CP70s by the caspase-9-initiated intrinsic pathway while being independent of the caspase-8-initiated extrinsic pathway.

In addition to the damage to the mitochondrial transmembrane potential and releasing caspase activators cytochrome c, participation of the Bcl-2 family of proteins is a vital event in the intrinsic apoptotic pathway [31]. Thus, we further studied the influence of PTFSs on the Bcl-2 family in ovarian cancer cells, including the pro-apoptotic proteins Bax and Bad, anti-apoptotic proteins Bcl-2 and Bcl-xL. On the other hand, for the extrinsic pathway, tumor necrosis factor-related apoptosis-inducing ligand (TRAIL), including Apo2L/TRAIL and Fas ligand (Fas L), would engage their respective death receptors, DR4/DR5 or Fas [55,56]. Thus, we checked the DR5, Fas L and Fas to further confirm if extrinsic apoptotic pathway signaling participated in PTFS-stimulated apoptosis. As depicted in Figure 5, PTFSs significantly upregulated Bax and Bad proteins, reduced Bcl-xL and Bcl-2, and had no influence on that of DR5, Fas L and Fas. These results suggest that PTFSs induced apoptosis through Bcl-2 family proteins linked to the intrinsic pathway rather than the extrinsic pathway in A2780/CP70s.

p53 is a multifunctional tumor suppressor that governs gene expression, cell cycle arrest, DNA repair, apoptosis, oxidative stress glucose metabolism and angiogenesis [37,57]. It has been reported that p53 stimulates apoptosis through the intrinsic pathway by regulating the Bcl-2 family and caspase family of proteins [37]. The association of cisplatin sensitivity, apoptotic induction and p53 pathway alterations is of popular interest in ovarian cancer therapy [29,58,59]. A number of prior studies in vitro and in vivo have shown that p53 alterations were associated with the failure of radiotherapy and chemotherapy in a range of cancers through loss-of-function, dominant-negative activity, or gain of oncogenic function. Additionally, many reports have focused on the p53-dependent anti-cancer effect of natural products [43,58,59,60,61,62]. Recently, it has been reported that platinum chemotherapy could induce additional mutations in *TP53* and further increase platinum resistance in ovarian cancer [63]. In this study, we suggested that p53 was up-regulated and phosphorylated at Ser 15 by PTFSs and played a vital function in intrinsic apoptosis stimulated through PTFSs. Overall, the results are in agreement with previous reports [29,58,59]. 

We further evaluated the effect on other proteins related with DNA damage such as ATM, p-ATM, Chk2, p-Chk2, and p-Histone H2A.X. A large number of serine and threonine residues of p53 protein are phosphorylated by many different protein kinases [64,65,66]. This is, in part, due to the response process from DNA damage to the p53 protein. Activation of ATM by autophosphorylation at Ser 1981 takes place as a response to exposed DNA double-stranded breaks, which is a sensor of DNA damage [67]. Chk2 acts downstream of ATM and is phosphorylated at Thr68 by ATM in responsive to DNA damage [68,69]. Rapid phosphorylation of histone H2A.X at Ser139 by ATM could result from DNA damage [70,71]. ATM phosphorylates p53 at Ser15, impairs the capability of MDM2 to bind p53 and encourages the buildup and stimulation of p53 as a response to DNA damage [42,72]. Our study shows that PTFSs increase the phosphorylation of ATM at Ser1981, histone H2A.X at Ser139, and Chk2 at Thr68 (Figure 7), which suggests that a DNA double-stranded break developed in ovarian cancer cells. Overall, p53 proteins and phosphorylation at Ser15 were significantly heightened in a concentration-dependent way (Figure 6a,b). Prior analyses have indicated that stimulation of ATM/Chk/p53 axis may encourage apoptosis in human pancreatic cancer cells [73] and in ovarian cancer cells [59]. These data suggest that this pathway could be a part of PTFS-stimulated ovarian cancer cell apoptosis.

Kitagawa et al. reported that Chakasaponin I, Chakasaponin II and Floratheasaponin A, which were extracted from tea flowers, could inhibit the proliferation of human digestive tract carcinoma cell line HSC-2, HSC-4, MKN-45 and Caco-2 cells with the IC_50_ being 4.4–40.6 µM, and could induce apoptosis and cell cycle arrest in HSC-2 cells [26]. Our previous paper [29] reported that saponins extracted from tea flowers, which contain 14 triterpenoid saponins exclusive of Chakasaponin I and Chakasaponin IV, could efficiently induce apoptosis in ovarian cancer cells via both intrinsic and extrinsic apoptotic pathways and also induce cell cycle arrest. The present study is the first evidence of anti-ovarian cancer function of Chakasaponin I in a relatively high purity. All three studies demonstrated the anti-cancer functions of tea flower saponins, and suggested that inducing apoptosis might be one of important underlying mechanisms. In addition, the present study reveals that it is through the intrinsic but not the extrinsic pathway that PTFSs induce apoptosis, dependent on p53 in ovarian cancer cells, which might be a more specific and pivotal mechanism, and is unlike the results of our previous paper. This difference in apoptotic mechanisms might be due to the different saponins we used. It is reported that differences in saponin structure, which include the type, position, and number of sugar moieties attached by a glycosidic bond at different positions of the rings, can characteristically influence biological responses, especially for the antitumor activity [74]. More studies focused on the anti-cancer mechanisms using one specific monomer are needed to explore the structure–function relationship of saponins in the anti-cancer mechanisms. In addition, several papers have reported the anti-cancer functions of triterpenoid saponins via different mechanisms. Momordin Ic has been reported to suppress liver cancer cell invasion via COX-2 inhibition and PPARγ activation [75,76]. Saikosaponin D extracted from Radixbupleuri has been reported to induce apoptosis and to block autophagic degradation in breast cancer cells [77]. Raddeanin A extracted from *Anemone raddeana* has been reported to suppress the angiogenesis and growth of human colorectal tumor by inhibiting VEGFR2 signaling [78]. All the above mechanisms are worth exploring in future research for anti-cancer functions of saponins extracted from tea flowers.

As many saponins exhibit pharmacological properties, a review of the absorption, disposition, and pharmacokinetics of saponins had been done [79]. Recently, the pharmacokinetics and excretion studies of sapindoside B in rat were conducted and reported the pharmacokinetic profiles of sapindoside B in the range of 2.5 to 12.5 mg/kg dosage-dependently, and only 2% intravenous dose of sapindoside B was excreted in its parent form over 48 h [80]. More research is needed to explain the contradiction between the low bioavailability of saponins and their potential anticancer activity in vivo. New drug delivery systems for saponins may be an effective strategy for developing these compounds into medicinal products.

In conclusion, our results indicate that PTFSs have a strong and preferential inhibition of cellular proliferation on A2780/CP70s and OVCAR-3s compared to IOSE-364s via intrinsic apoptosis rather than the extrinsic pathway. We also found that p53 protein has a crucial function in intrinsic apoptosis induced by PTFS treatment, and downstream proteins like Bcl-2 families and caspase families were successively regulated. Additionally, these activities were at least partly induced by DNA damage through the ATM/Chk2/p53 axis. Our data help us to understand the mechanisms through which PTFSs may lead to preventing and treating platinum-resistant ovarian cancer. PTFSs were composed of Chakasaponin I and IV. Chakasaponin I may be a potential natural compound for treating platinum-resistant ovarian cancer, and more anti-cancer bioactivities of saponins of tea (*Camellia sinensis* (L.) *Kuntze*) flower are worth exploring in the future.

## 4. Materials and Methods 

### 4.1. Preparation of Purified Tea Flower Saponins (PTFSs)

Dried tea *(Camellia sinensis* (L.) *Kuntze*) flowers (Zhejiang Yilongfang Co., Quzhou, China) were ground and then extracted with 70% methanol through refluxing at 60 °C for 2 h. The methanol isolate was then divided into ethyl-acetate and n-butanol soluble layers. The n-butanol layer was vacuum concentrated, collected, underwent D101 column chromatography, and eluted through 0%, 15%, 30%, 45%, 60%, 75% and 90% ethanol-aqueous solution (*v/v*) at a rate of 2 bed volume (BV, 2.5 L)/h each for 1 BV. The 90% ethanol-eluted fraction was collected, concentrated and purified by reversed-phase preparative middle-low pressure liquid chromatography system (GE ÄKTA purifier100, Uppsala, Sweden) alongside a SinoChrom ODS-BP column (5 μm, 250 mm × 10.0 mm i.d., Elite, Dalian, China). Elution was conducted using a mobile phase composed of formic acid, water and acetonitrile (0.1:49.9:50.0, *v/v/v*) at 210 nm at 1.5 mL/min to acquire five fractions. The first one was additionally filtered using a Waters XBridge Shield RP18 column (Waters, Milford, MA, USA) to produce PTFSs. Mobile phase A included formic acid and water (0.1:99.9, *v/v*), and B included formic acid and acetonitrile (0.1:99.9, *v/v*). The elution gradient was 42% B for 13 min, 45% B for 20 min and 47% B for 15 min. The rate of the mobile phase was 1.5 mL/min. After separation, PTFSs were collected and measured by UPLC coupled with electrospray ionization quadrupole time-of-flight mass spectrometry (UPLC-Q-TOF/MS/MS), as previously explained [23]. PTFSs were dissolved in DMSO to prepare a solution with the final concentration of 20 mg/mL, and was kept at −20 °C DMSO was bought from Sigma-Aldrich (Sigma, St. Louis, MO, USA).

### 4.2. Cell Culture

A2780/CP70 and OVCAR-3, two human ovarian cancer cell lines, were provided by Dr. Jiang at West Virginia University. IOSE-364, a normal ovarian surface epithelial cell line, was kindly supplied by Dr. Auersperg at the University of British Columbia. Cell lines were maintained in RPMI 1640 with 10% fetal bovine serum (FBS) and 1% penicillin-streptomycin solution. Cells were maintained in an incubator with 5% CO_2_ at 37 °C RPMI-1640 and bovine serum albumin (BSA) was bought through Sigma-Aldrich (Sigma, St. Louis, MO, USA). Penicillin-streptomycin solution was bought through Thermo Scientific (Waltham, MA, USA). FBS and phosphate-buffered saline (PBS) were bought through Life Technologies (Invitrogen, Grand Island, NY, USA).

### 4.3. Cell Viability

The MTS assay was performed to measure cell viabilities of A2780/CP70, OVCAR-3 and IOSE-364. Briefly, cells were seeded in 96-well plates (1 × 10^4^ cells per well) for 24 h. Then, various dosages of PTFSs (0–3.5 µg/mL) were added with an equivalent volume of DMSO as control. Cells were washed twice with PBS after incubation for 24 h, and 100 μL of fresh Aqueous One Solution was placed into each well. After incubating at 37 °C for 1 h, the absorbance of cells was read at 490 nm using a microplate reader (BioTek, Winooski, VT, USA). Cell viability was expressed as a proportion of controls. CellTiter 96 AQueous One Solution Cell Proliferation Assay was bought through Promega (Madison, WI, USA).

### 4.4. Hoechst 33342 Staining

The degree of apoptosis present in cells was determined using Hoechst 33342 staining. In brief, A2780/CP70 and OVCAR-3 cells were placed into 96-well plates (1 × 10^4^ cells per well) for 24 h, and treated using PTFSs (0–3 μg/mL) with or without of 20 μM PFT-*α* for 24 h. Then, cells were cleaned with PBS and stained with Hoechst 33342 (10 μg/mL) for 15 min in the dark at 37 °C. Fluorescence was observed by microscopy (ZEISS, Heidelberg, Germany). Hoechst 33342 and PFT-α were bought through Sigma-Aldrich (Sigma).

### 4.5. Evaluating Mitochondrial Membrane Potential

A2780/CP70s were seeded in black 96-well plates (1 × 10^4^ cells per well) for 24 h, then treated with PTFSs (0, 1, 2, 3, 4 μg/mL) for 24 h, washed twice using PBS, and incubated for 30 min with 10 μg/mL JC-1 dye solution. Fluorescence was quantified at an excitatory emission ratio of 485/590 for red aggregates and 485/535 for green monomers through the use of a fluorescence plate reader (BioTek, Winooski, VT, USA).

### 4.6. Apoptosis Analysis by Flow Cytometry

A2780/CP70s and OVCAR-3s were seeded in 60 mm plates (1 × 10^6^ cells/dish). After cells adhered for 24 h, various doses of PTFSs (0, 1, 2, 3 μg/mL) were added for another 24 h and harvested for additional studies. For apoptosis examination, an Alaxa Fluor 488 Annexin V/Dead Cell Apoptosis kit (Thermo Scientific, Waltham, MA, USA) was utilized. Treated cells were harvested and cleaned twice using cold PBS, which was followed by the resuspension of cells in PI and annexin V buffer, as per the manufacturer’s established protocol. Samples were assessed utilizing a FACSCaliber flow cytometry system (BD Biosciences, San Jose, CA, USA).

### 4.7. Cellular Caspase Activity Assay

A2780/CP70s were seeded into 96-well plates (1 × 10^4^ cells per well). After cells adhered for 24 h, different concentrations of PTFSs (0–3 μg/mL), with or without 20 μM PFT-α, were added and the cells were cultured for another 24 h. An equivalent volume of DMSO was added to the control groups. Caspase-Glo-3/7, -8 and -9 (Promega, Madison, WI, USA) regents were supplemented and incubated for 30 min. Luminescence was measured through the use of a microplate reader (BioTek, Winooski, VT, USA). Analyses were performed three times and results were conveyed as a proportion of controls.

### 4.8. Small Interfering RNA (siRNA) Transfecting

A2780/CP70s were seeded into 60 mm dishes (1 × 10^6^ cells/dish), incubated overnight, and then transfected with p53 siRNA (Santa Cruz, Dallas, TX, USA) using Lipofectamine 2000 transfection reagent (Invitrogen, Grand Island, NY, USA) as per the manufacturer’s established guidelines. Cells transfected using control siRNA (Santa Cruz, Dallas, TX, USA) were utilized as controls. Post-24 h transfection period, cells were treated with or without PTFSs (3 μg/mL) for 24 h. Then, lysates were harvested for western blotting analysis.

### 4.9. Western Blotting

Proteins were determined by specific monoclonal antibodies in Western blot analysis. PTFSs were added to A2780/CP70s and OVCAR-3s, with or without 20 μM PFT-α or p53 siRNA, for 24 h as previously shown. Protein was separated using Mammalian Protein Extraction Reagent and 1% protease inhibitor cocktail. As per our prior study [29], the total protein concentration was measured by a BCA protein assay kit (Pierce, Rockford, IL, USA). Equivalent levels of protein were loaded onto SDS-PAGE and transferred onto nitrocellulose membranes. The membrane was placed in blocking buffer at room temperature for 1 h, and incubated with targeted primary antibodies at 4 °C overnight. The next day, membranes were incubated with secondary antibodies. After washing, the protein was visualized using the ChemiDocTM MP System (Bio-Rad, Hercules, CA, USA). GAPDH was used to normalize relative values of each protein. Primary antibodies targeting Apaf-1, Bax, Bcl-XL, Bcl-2, cytochrome c, cleaved caspase 3, caspase 3, caspase 7, caspase 8, caspase 9, DR5, FasL, Fas, p53, p-p53 (Ser15), PARP, PCNA, p-Histone H2A.X(Ser139), MDM2 and secondary antibodies were bought through Cell Signaling Inc. (Danvers, MA, USA). Primary antibodies targeting p-Chk2 (Thr68), Chk2 (H-300), Bad, and GAPDH (0411) were bought from Santa Cruz Biotechnology (Dallas, TX, USA).

### 4.10. Statistical Analysis

The data were exhibited as mean ± standard error of mean (SEM) from at least 3 independent experiments, and at least 3 biological replications for each independent experiment have been done. Data were assessed by SPSS (SPSS, Chicago, IL, USA) using the Shapiro–Wilk normality test, one-way analysis of variance (ANOVA) and post-hoc test (2-sided Dunnett’s test). *p*-values < 0.05 represent statistical significance.

## Figures and Tables

**Table 1:** Mass spectrometry data in negative mode for saponins identified from PTFSs.

Peak	Retention Time (Min)	[M-H]^−^	MS^2^	Formula	Peak Identity	%
1	50.52	1173.5714	1042,993,909,569	C_57_H_90_O_25_	Chakasaponin IV	8.1
2	51.57	1215.5828	1083,1035,951,933,611	C_59_H_92_O_26_	Chakasaponin I	80.1
3	62.43	1215.5949	1083,1035,951,933,611	C_59_H_92_O_26_	Floratheasaponin A	1.4

## Abbreviations

BSA	Bovine serum albumin
BV	Bed volume
CC3	Cleaved caspase 3
CC9	Cleaved caspase 9
C-PARP	Cleaved PARP
F-PARP	Full length PARP
cyto c	Cytochrome c
DMSO	Dimethyl sulfoxide
FBS	Fetal bovine serum
PBS	Phosphate-buffered saline
PFT-α	Pifithrin-α
PI	Propidium iodide
PTFSs	Purified tea *(Camellia sinensis)* flower saponins
SEM	Standard error of mean
